# A Soft Computing Based Approach Using Modified Selection Strategy for Feature Reduction of Medical Systems

**DOI:** 10.1155/2013/587564

**Published:** 2013-03-21

**Authors:** Kursat Zuhtuogullari, Novruz Allahverdi, Nihat Arikan

**Affiliations:** ^1^Department of Electronic and Computer Education, Technical Education Faculty, Selcuk University, Selcuklu, 42003 Konya, Turkey; ^2^Department of Computer Engineering, Faculty of Technology, Selcuk University, 42003 Konya, Turkey; ^3^Department of Urology, Ankara University Faculty of Medicine, 06100 Ankara, Turkey

## Abstract

The systems consisting high input spaces require high processing times and memory usage. Most of the attribute selection algorithms have the problems of input dimensions limits and information storage problems. These problems are eliminated by means of developed feature reduction software using new modified selection mechanism with middle region solution candidates adding. The hybrid system software is constructed for reducing the input attributes of the systems with large number of input variables. The designed software also supports the roulette wheel selection mechanism. Linear order crossover is used as the recombination operator. In the genetic algorithm based soft computing methods, locking to the local solutions is also a problem which is eliminated by using developed software. Faster and effective results are obtained in the test procedures. Twelve input variables of the urological system have been reduced to the reducts (reduced input attributes) with seven, six, and five elements. It can be seen from the obtained results that the developed software with modified selection has the advantages in the fields of memory allocation, execution time, classification accuracy, sensitivity, and specificity values when compared with the other reduction algorithms by using the urological test data.

## 1. Introduction

 The information based systems consisting high input spaces require high processing times and memory usage. Feature reduction algorithms are used for determining the dominant and significant attributes for representing the whole data with no or minimum information loss. Reduction systems aim to reduce the computation times and prevent information storage problems when processed with artificial intelligence techniques. Rough sets theory is very significant in data mining and is used for input attribute selection purposes for representing the whole data set [1, 2]. The attribute selection algorithms aim to explore and analyze the hidden data that are embedded in the information based systems and these algorithms make the data processible by soft computing methods [3].

 Most of reduction algorithms that use rough sets based reduction algorithms have the problems of input space limits and high memory demand problems [2]. The decision relative discernibility matrix function approach has the restrictions in the numbers of the attributes and requires high memory and time demand when used in the software systems. These problems give rise to memory errors when the systems with high input spaces are processed by the attribute reduction algorithms and cause input space restrictions. The object related discernibility based approach of Johnson algorithm has less classification accuracy when tested with artificial neural network classifier. In most of the reduction systems, information loss in feature selection system is also a significant problem. 

 The different versions of rough sets methodologies are used for data mining based systems for reduction and knowledge discovery purposes. In the data mining and knowledge discovery systems, the input database is represented by the inputs named as attributes. Each column of information system represents attributes and each row represents a case or an event. Hidden data embedded in the knowledge based systems are investigated by the data mining based systems. The significant input attributes are determined by the rough sets based methodologies. The data mining based procedures are useful to overcome the problems caused by high dimensional data. The data mining based procedures help the data to be classified by artificial intelligence based system like artificial neural network classifiers. The different versions of feature selection algorithms are used for clustering and data mining purposes [3]. The reducing of number of the input attributes and selecting dominant features that represent the database are made for processing the data efficiently by soft computing based methods [3–5]. This procedure is significant for knowledge based systems because the processing database that consists of high inputs takes longer processing times or causes memory errors in software systems. The rough sets theory has a significant role in the feature reduction mechanisms of the knowledge based systems. The theory has found applications in many domains, such as decision support, engineering, environment, banking, medicine, and other information based systems. Rough set methodology is based on the theory that every object of the universe of discourse is related with knowledge. Objects which are characterized by the same information are indiscernible (similar) in view of the available information about them.

 An information system is expressed as *S* = (*U*, *A*), where *U* and *A* represent nonempty sets (the universe) and the set of attributes, respectively. An information system can be separated into two attribute groups. These input features of the input space are called the conditional attributes and represented by “*C*” and the output of the system is called the decision attribute and represented by “*D*” [4–6].

 The genetic algorithms are the computational models used for generating solutions for specified areas and use the solution candidate models that are named as chromosomes. The genetic algorithm based strategies explore the solution candidates by constructing the generations. These algorithms apply recombination operators and mutation operators to these structures to obtain critical solution candidates. Crossover methods are applied to the selected chromosomes for obtaining different solution candidates. These optimization algorithms evaluate the potential solutions and produce new solutions for finding the optimal solution. Selection algorithms are used for obtaining the generation that is used for crossover and mutation operators. The goodness of a solution is represented typically by the fitness value and calculated according to the specific problem. 

 The genetic algorithm based models propose the advanced solution techniques for calculating the optimal results by producing new solution candidates. These soft computing methods aim to find the better solutions by applying genetic algorithm operators like selection, crossover, and mutation. These operators are the computational mathematical models for finding the optimal solutions for the investigated problem. An implementation of a genetic algorithm begins with a population of chromosomes. Then the genetic algorithm based system uses the genetic algorithm operators for finding better solutions. In a broader usage of the term, a genetic algorithm is any population based model that uses selection and recombination operators to generate new sample solution points in a search space.

 The aim of the study is to construct a reduction software that supports large input numbered systems with effective memory usage and processing time. Locking to local solutions and high computation times is also a problem in the genetic algorithm selection mechanisms like roulette wheel selection and some of the other selection strategies. These problems have been solved by using the developed hybrid software using new proposed modified selection that is based on artificial selection system and faster and efficient reducing is obtained by optimum memory usage. The developed software has the capability of finding the reducts (reduced input attributes) more faster and efficiently and the locking to the local solutions problem is also solved in the designed modified artificial selection algorithm. In Section 2, constructed feature reduction software using genetic algorithm with new modified selection and rough sets (FRSGR) is expressed. In Section 3, results and discussions and in Section 4, conclusions are given.

## 2. Materials and Methods

### 2.1. Features of Genetic Algorithm and Rough Sets Based Hybrid Attribute Selection Software

In this study, feature reduction software using genetic algorithm with new modified selection and rough sets based hybrid system (FRSGR) has been developed. Delphi 7 programming language has been used for designing the interface of FRSGR. In the designed system, a new modified selection system that depends on artificial selection method is proposed and used. The developed system not only supports the medical systems but also the information systems with high dimensional input spaces. 

 In the constructed software genetic algorithm system using new modified selection system is integrated with rough sets attribute reduction system for finding the optimal reducts (reduced input attributes for representing the whole data) of the medical and information based systems with high input spaces. Attribute dependency value of rough sets methodology is used as the fitness value for the genetic algorithm based solution candidate generation system. The software can be stopped according to the fitness value or the maximum number of generations determined by the user of the software. A new selection mechanism based on artificial selection algorithm is designed for the genetic algorithm part of the software. In the FRSGR, roulette wheel and modified artificial selection algorithms are used. Linear order crossover algorithm is used as the recombination operator in the genetic algorithm part of the constructed software. Arbitrary two input change and three input change methods are used as the mutation operators. In addition, another software based on decision relative discernibility matrix is also constructed by using Delphi programming language as a test software for comparing the performance of the designed FRSGR. The new selection mechanism designed (proposed) and used in the system is the modified version of the artificial selection algorithm. The modified version decreases the computation time when compared with the classical approach and roulette wheel mechanism and finds the solution candidates effectively by preventing the locking to local solution candidates. Better results are obtained when compared with the classical artificial selection and roulette wheel selection mechanisms.

 In the roulette wheel selection mechanism, the larger regions are assigned to the chromosomes with larger fitness values. The chromosomes with smaller fitness values have small regions. This strategy selects a random point in the region. The chromosomes with higher fitness values can be selected more frequently because the probability of selection of larger region is higher. In the roulette wheel selection strategy of FRSGR, the separated regions are determined by the fitness value determined by the rough sets based strategy. In the FRSGR, the fitness value for roulette wheel selection mechanism is determined by the attribute dependency value of the solution candidate. 

 Rough set is itself the approximation of a set by a pair of precise concepts named as lower and upper approximations [3]. The lower approximation expresses the domain of objects that are known with certainty to belong to the subset of interest, whereas the upper approximation is a description of the objects that possibly belong to the subset. *I* = (*U*, *A*) represents a knowledge based system, where *U* is a the nonempty set of finite objects and *A* is a nonempty finite set of attributes such that *a* : *U* → *V*_*a*_ for every *a* ∈ *A*. *V*_*a*_ represents the set of values that attribute a may take. *A* = {*C* ∪ *D*}, where *C* is the set of input features and *D* is the set of class indexes for classification purposes in decision based systems [3].

 The lower approximation and upper approximation concepts are expressed by (1). *X* can be approximated using the information in *P* by constructing the *P*-lower and *P*-upper approximations of the classical crisp set *X* as follows:

(1) $$\begin{array}{c} \underline{P}X=\left\{x\;|\;{\left[x\right]}_{P}\subseteq x\right\},\\ \overset{-}{P}X=\left\{x\;|\;{\left[x\right]}_{P}\;\cap x\neq \phi \right\}. \end{array}$$

The positive, negative, and boundary regions of the rough sets are expressed by [3–6]

(2) $$\begin{array}{c} \text{PO}{\text{S}}_{P}\left(Q\right)=\;\underset{}{\overset{}{\underset{x\in U/Q}{⋃}}\underline{P}X,}\; \end{array}$$

(3) $$\begin{array}{c} \text{NE}{\text{G}}_{P}\left(Q\right)=U-\underset{}{\overset{}{\underset{x\in U/Q}{⋃}}\overset{-}{P}X,} \end{array}$$

(4) $$\begin{array}{c} \text{BNP}\left(Q\right)=\underset{}{\overset{}{\underset{x\in U/Q}{⋃}}\overset{-}{P}X}\;-\underset{}{\overset{}{\underset{x\in U/Q}{⋃}}\underline{P}X.} \end{array}$$

Attribute (feature) dependency values of rough sets methodology are used for the fitness value of the generated candidates in FRSGR. The constructed software uses the feature dependency value of rough sets methodology for each chromosome for finding the optimal reducts with high performance. In the rough sets theory, feature dependency value is the ratio of the positive region to the solution space and is expressed in (5). A set of attributes *Q* depends on a set of attributes *P* and for *P*, *Q* ⊂ *A*, *Q* depends on *P* in a degree *k* (0 ≤ *k* ≤ 1) and is denoted by *P*⇒*kQ*.

 As a stopping criterion and attribute evaluation mechanism, *α* threshold level is used in the FRSGR and this value can be determined by the user in the developed system. In the FRSGR user defined the attribute dependency value is accepted as the stopping criterion and threshold level for the stopping criterion. 

 The stopping criterion for the developed hybrid system is accepted as a threshold level for attribute (feature) dependency value which is calculated by the proportion of the number of the elements in the positive region to the elements in the universal set and shown in (6).

 Selection systems are significant for genetic algorithm based systems [7, 8]. In the classical artificial selection algorithm, the last two generations are used for constructing the gene pool and the best and worst valued chromosomes are selected for constructing the intermediate generation that is used for crossover and mutation operators. In the developed new modified selection mechanism based on artificial selection, initially, the first two generations are generated randomly and these generations are combined for constituting the first gene pool. This modification prevents the repetitions of the solution chromosomes. The second and the following generations use the last two generations like the classical approach and the algorithm continues iteratively. The chromosomes are ordered according to the attribute dependency value of FRSGR in the gene pool. In the modified new proposed version, the intermediate generation is selected from the best valued, middle valued, and the worst valued chromosomes with the desired percentage values determined by the user of the software. The middle region chromosomes are selected from the region starting from the middle fitness valued point towards the worst solution candidates in the gene pool when solution candidates are ordered in the descending order in the gene pool. The chromosomes are selected by the middle region by obeying the order numbers of the chromosomes.

 In contrary with the classical approach, the middle fitness valued chromosomes are added to the best and worst valued chromosomes. The modifications give rise to faster reduct (reduced input attributes) obtaining.

 The abbreviation “sol. can.” denotes the “solution candidate” that is used for the chromosomes in the generation. The solution chromosomes that are equal or higher than *α* threshold level are accepted as the results. In the modified artificial selection algorithm proposed in this study, the solution addition type (percentage of chromosomes selected from intermediate part) added to the algorithm is calculated by (7). The developed system also supports the classical version. In the classical version, best and worst solutions selected from the last two generations are used for generating the gene pool. But in the modified version used in the developed software, the first two starting generations are constructed randomly, and the last two generations are used in the following steps. Best, worst, and middle valued chromosomes are selected in the desired percentages. 

 In the FRSGR, the chromosomes of the last two generations are listed in the descending order according to their attribute dependency values. The middle region proposed in the modified version starts from the middle point of the list continues downwards. The “Mid. Sol.” term is used for the abbreviation of “solution candidates (chromosomes) from middle region” and “Best Sol.” and “Worst Sol” are used as the abbreviations named as the best solution candidates and worst solution candidates, respectively.

 This modification decreases the computation time and prevents the algorithm to be locked in the local solution points. The percentage values of the selected chromosomes from middle region are named as solution addition percentage in the developed software interface and expressed by the abbreviation “Mid. Sol.%” and shown in (7)

(5) $$\begin{array}{c} k=\gamma_{P}\left(Q\right)=\frac{\left|\text{PO}{\text{S}}_{P}\left(Q\right)\right|}{\left|U\right|}, \end{array}$$

(6) $$\begin{array}{c} \text{for}\;\text{If}\;\gamma_{P}\left(Q\right)\;=\frac{\left|\text{PO}{\text{S}}_{P}\left(Q\right)\right|}{\left|U\right|}\;\text{of}\;\text{the}\;\text{sol}.\;\text{can}.\;\geq \alpha \;\text{then}\;\text{stop}, \end{array}$$

(7) $$\begin{array}{c} \text{Mid}.\;\text{Sol}.\%\;+\;\text{Best}\;\text{Sol}.\%+\;\text{Worst}\;\text{Sol}.\%=100\%. \end{array}$$

The reduction procedure also decreases the training times of the artificial neural network classifier system. Delphi programming language and interface have been used for developing FRSGR and variable input artificial neural network test software that uses back propagation algorithm.

 The constructed software is generated with the adaptation of the multiple input databases and the selection method is used for determining the gene pool for the crossover and mutation operators of the genetic algorithm. Linear order crossover method is used as a recombination operator. Falkenauer and Bouffouix proposed a modified version of order crossover, the linear order crossover (LOX) [9–11]. The working principle of LOX is described below.

- Random points are selected from the parent chromosomes for determining sublists. The random points for crossover can be started from different locations in the parent chromosomes but the lengths of the sublists are accepted as the same.
- Interchange the sublists taken from the parents with the holes previously defined.
- Prevent the repetitions in the chromosome genes preserving the orders in the parent chromosomes and fill the left and right side of the crossover points of chromosome using the genes taken from the parent.

Mutation operators are used in the system because high crossover rates are used in the developed system that gives rise to generated different solution candidates. Arbitrary two input change and three input change methods are used as the mutation operators. The random selected two inputs are changed in arbitrary two input change methods and random selected three inputs are changed in the arbitrary three input change methods [12–14].

 By using FRSGR, high classification accuracy, sensitivity, specificity, PPV and NPV values have been obtained when neural network classifier has been used and the processing times were reduced by using this selection algorithm and input number restriction problems of most of the reduction algorithms were solved. In the genetic algorithm based systems, locking to the local solutions is also a serious problem that increases the computation times and prevents searching the solution spaces for finding the optimal solutions. This problem is also solved by the developed modified artificial selection system by generating the first two startup generations randomly and using not only the best and worst solution candidates but also the chromosomes with middle (intermediate) valued attribute dependency values. 

**Figure 1:**
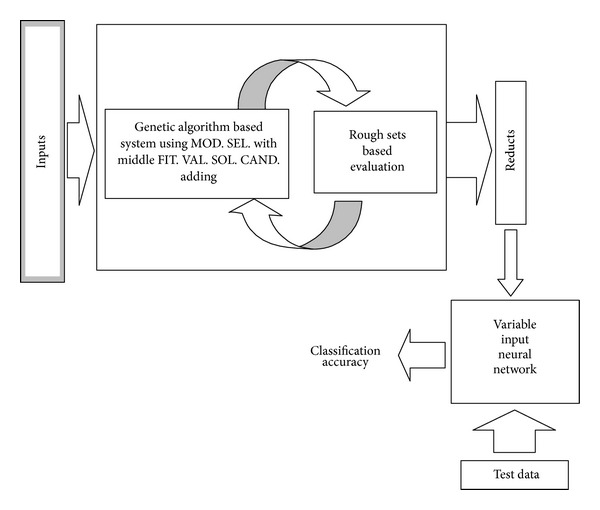
General structure of the developed software.

**Figure 2:**
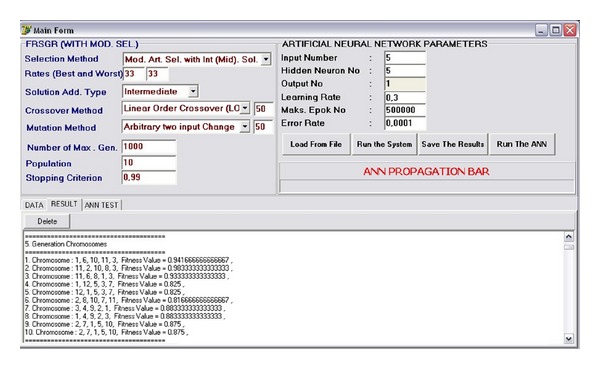
General structure of software interface (FRSGR).

**Figure 3:**
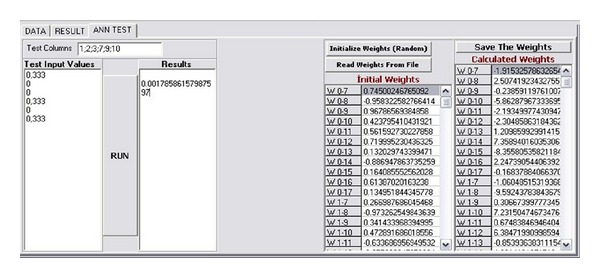
Artificial neural network and test part of the developed software.

**Figure 4:**
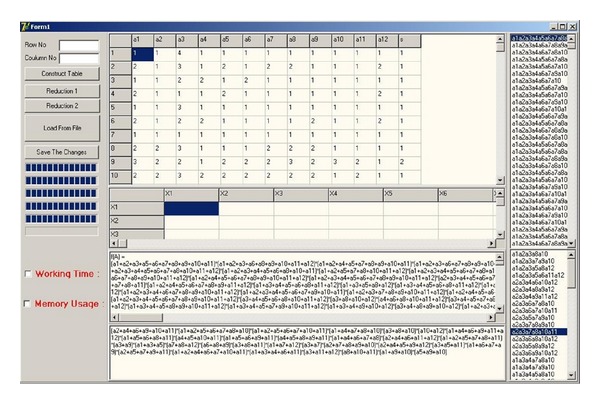
Another software developed for reducing the input attributes by using the decision relative discernibility approach.

 An artificial neural network (ANN) software is constructed and added to the output of the reduct generation system. The general structure of the generated software is shown in Figure 1. In the ANN part of the software, backpropagation based classifier is used and the number of inputs, hidden neurons, and learning rate can be adjusted by the user. The developed ANN software has the capability to train the selected columns determined by the reducts of the FRSGR.  Figure 2 shows the designed software interface and Figure 3 shows the ANN part of the software. In the new proposed modified artificial selection method, the first two starting generations are constructed randomly and the best, worst, and middle fitness valued chromosomes are used for constructing the gene pool in the modified selection whereas in the classical version only the best and worst fitness valued chromosomes are used. In the following generations the last two generations are used for the gene pool like the classical approach.  Figure 4 shows another software developed for reducing the input attributes by using the decision relative discernibility approach for comparing the results. 

 Another software using decision relative discernibility matrix and function based reducing mechanism is constructed for comparing the performance with the designed FRSGR. The decision relative discernibility based reduction software is accelerated and optimized for comparing the performance with FRSGR. Discernibility matrix based system uses Boolean algebra and set theory for the obtaining reducts for representing the whole medical system.

 A discernibility matrix is expressed by using a decision table (*U*, *C* ∪ *D*) which is a symmetric |*U* | ×|*U*| matrix and the discernibility functions can be calculated by using this matrix and approach. A discernibility function *f*_*d*_ is a Boolean function of m Boolean variables *a*_1_^  ∗^,…, *a*_n_* (corresponding to the attributes named as *a*_1_,…, *a*_*n*_ from a given entry of the discernibility matrix). The discernibility function is expressed in (8). *f*_*d*_ represents the discernibility function and *i* and *j* are the indexes used for the matrix cells [3, 4] as follows:

(8) fd(a1    ∗,…,am    ∗)=∧{∨Cij ∣ 1≤j≤i≤|U|,     Cij≠∅}.

The results obtained from the FRSGR are compared with the Johnson algorithm based reducer of Rosetta software. The Johnson based reducer derives the reducts by using the a variation of Greedy algorithm. This algorithm has a natural bias towards finding a single prime implicant of minimal length. The reduct named as “*B*” is found by running the algorithm expressed below. The *S* denotes the set of sets corresponding to the discernibility function and *w*(*S*) shows a weight for set *S* in *S* that automatically computed from the data. Support for computing approximate solutions is provided by aborting the loop when “enough” sets have been removed from *S*, instead of requiring that *S* has to be fully emptied [15, 16].

1. *B* = *∅*.
2. *a* expresses the attribute that maximizes ∑*w*(*S*) where the sum is taken over all sets *S* in *S* that include *a*. The ties are resolved arbitrarily.
3. *a* is added to *B*.
4. All sets *S* from *S* that include *a* are removed.
5. If *S* = *∅*; *B* is returned, if not, go to step 2.

Uroflowmetry is a diagnostic test that is made for checking for abnormalities in the flow rate of a patient's urine. Uroflowmetric measurements are very important for determining the urological illnesses like kidney problems, urethral obstructions, urethral strictures, abnormal bladder activities, prostatic diseases, and bladder obstructions. The volume of urine left in the bladder is measured after uroflowmetry test. The residual urine volume that cannot be voided by the bladder shows the volume of urine left in the bladder after the test. Voiding time shows the time passed for voiding the bladder and measured by the devices of uroflowmetry [17–19]. 

 The FRSGR generates solution candidates with multiple input variables and can produce solution candidates with desired input number range. The system has 12 input variable, and 1 classification variable (decision variable). The input variables of the system are uroflowmetric measurements named as maximum flow rate (mlt./s), average flow rate (mlt./s) and residual urine volume (mlt.) and the sampled flow rate values (mlt./s) from the uroflowmetry graph in the period of *T*/4 and 3*T*/4. *T* represents the voiding time and the maximum flow rate is denoted by the input variable named as “*a*_1_”; average flow rate and residual urine volume are represented by the *a*_2_ and *a*_3_, respectively.

 The input variables *a*_4_, *a*_5_, *a*_6_, *a*_7_, *a*_8_, *a*_9_,…, *a*_12_ (9 sampled uroflowmetric values) represent the sampled flow rate measurements in the period of *T*/4 and 3*T*/4 in the uroflowmetry graph. The period of *T*/4 and 3*T*/4 is divided into 9 parts in the uroflowmetry test for getting the sampled flow rate values. The maximum flow rate is expressed by 4 linguistic variables named as very low, low, medium, and high and are expressed by 1, 2, 3, and 4 in Table 1. The average flow rate is expressed by 4 linguistic variables very low, low, medium and high and encoded by 1, 2, 3, and 4, respectively. The accepted reference values and threshold levels are given below. The same threshold levels for the average flow rate values are accepted for the threshold values of the sampled flow rate values. The residual urine volume is denoted by none, medium, high, and very high and denoted by the numbers 1, 2, 3, and 4, respectively. The sampled flow rate values (*a*_4_, *a*_5_, *a*_6_, *a*_7_, *a*_8_, *a*_9_,…, *a*_12_) are denoted by four linguistic variables named as very low, low, medium, and high and denoted by 1, 2, 3, and 4, respectively. 

Maximum Flow rate (mlt./s)

 1—Very Low: 0 mlt./s ≤ *x* < 10 mlt./s
 2—Low: 10 mlt./s ≤ *x* < 20 mlt./s
 3—Medium: 20 mlt./s ≤ *x* < 30 mlt./s
 4—High: 30 mlt./s ≤ *x* ≤ 40 mlt./s

Average Flow rate (mlt./s)

 1—Very Low: 0 mlt./s ≤ *x* < 7 mlt./s
 2—Low: 7 mlt./s ≤ *x* < 14 mlt./s
 3—Medium: 14 mlt./s ≤ *x* < 25 mlt./s
 4—High: 25 mlt./s ≤ *x* ≤ 40 mlt./s

Residual Urine Volume (mlt.)

 1—None: 0 mlt
 2—Medium: 0  mlt.<*x* < 50 mlt.
 3—High: 50 mlt. ≤ *x* < 150 mlt.
 4—Very High: 150 mlt.≤*x* ≤ 500 mlt.

Some of the transactions in the medical (urological) database are shown in Table 1. The database consists of 120 transactions. Each transaction (row) denotes the patients and each column represents the urological measurements. The database is taken from the patient database and constructed by the help of urology expert. The classification attribute determines the very risky, risky, and healthy groups according to the uroflowmetric measurements and residual urine volume. The classification attribute is expressed by the letter “*d*” and three linguistic variables named as very risky, risky, and healthy and symbolised by the numbers 1, 2, and 3, respectively.

### 2.2. Artificial Neural Network Classifier

 The artificial neural network (ANN) software with variable input processing feature is constructed by visual programming language. The output of the FRSGR is attached to developed flexible artificial neural network classifier software and the dominant attributes representing the data sets (reducts) are accepted as the input variables. The number of input variables and the hidden neurons, the error rate and the learning rate variables can be determined by the user of the interface. Back propagation method is used in classification software. Calculated weights can be saved to the text files and read from them for faster processing purposes. Output value of ANN is calculated by forward propagation. Updating of the weights is made in the backward propagation phase. Net and output values for middle layer neurons are calculated by (9) when Sigmoid function is used. *C*_*j*_ represents the output value of middle neuron [20, 21] as follows:

(9) $$\begin{array}{c} \text{NE}{\text{T}}_{j}=\sum_{i=1}^{i=n}X_{i}W_{ij},\\ C_{j}=\frac{1}{1+e^{-(\text{NE}{\text{T}}_{j}^{a}+\beta_{j}^{a})}}. \end{array}$$

In the backward propagation algorithm the initial weights are updated according to the position of the neurons. The updated weights are applied to the next iteration. Updating of the weights between the middle and output layer is made by using (10)–(13). In the equations below, *λ* expresses learning constant and *α* represents the momentum coefficient. *β* represents the bias weights and the Δ*β* represents the change of the weights of the biases. *C*_*m*_ represents the output value of the output neuron and *C*_*j*_ represents the output value of the middle neuron [20–24].

 In (10), (13), and (14), Δ*A*_*jm*_^*a*^ represents the change in the weight between middle and output neuron as follows:

(10) $$\begin{array}{c} \Delta {A_{jm}^{a}}_{}^{}\left(t\right)=\lambda \delta_{m}C_{j}^{a}+\alpha \Delta A_{jm}^{a}\left(t-1\right), \end{array}$$

(11) $$\begin{array}{c} \delta_{m}=f^{\prime }\left(\text{NET}\right)\cdot E_{m}, \end{array}$$

(12) $$\begin{array}{c} \delta_{m}=C_{m}\left(1-C_{m\;}\right)E_{m}. \end{array}$$

The new values of the weights are calculated by (13)–(15). The weights of the bias neurons are updated using (14)-(15). *A*_*jm*_ represents the weights between the middle layer and the output layer and Δ*A*_*jm*_^*a*^ represents the change in the weight of *A*_*jm*_. In the equations used for updating the weights of backpropagation network, *k* is an index used for representing the input layer, *j* denotes the middle layer, and *m* represents the output layer:

(13) $$\begin{array}{c} A_{jm}^{a}\left(t\right)=A_{jm}^{a}\left(t-1\right)+\Delta A_{jm}^{a}\left(t\right), \end{array}$$

(14) $$\begin{array}{c} \Delta \beta_{m}^{ç}\left(t\right)=\lambda \delta_{m}+\alpha \Delta \beta_{m}^{ç}\left(t-1\right), \end{array}$$

(15) $$\begin{array}{c} \beta_{m}^{ç}\left(t\right)=\beta_{m}^{ç}\left(t-1\right)+\Delta \beta_{m}^{ç}\left(t\right). \end{array}$$

In the update phase of the weights between the middle layer and the input layer, [20–24] the following is:

(16) $$\begin{array}{c} \Delta A_{kj}^{i}\left(t\right)=\lambda \delta_{j}^{a}{Ç}_{k}^{i}+\alpha \Delta A_{kj}^{i}\left(t-1\right),\\ \delta_{j}^{a}=f^{\prime }\left(\text{NET}\right)\sum_{m}\delta_{m}\;A_{jm}^{a},\\ A_{kj}^{i}\left(t\right)=A_{kj}^{i}\left(t-1\right)+\Delta A_{kj}^{i}\left(t\right),\\ \Delta \beta_{j}^{a}\left(t\right)=\lambda \delta_{j}^{a}+\alpha \Delta \beta_{j}^{a}\left(t-1\right),\\ \beta_{j}^{a}\left(t\right)=\beta_{j}^{a}\left(t-1\right)+\Delta \beta_{j}^{a}\left(t\right). \end{array}$$

In the neural network classifier system, normalization procedure is made according to the values in the columns. The Normalization equation used in the procedure is expressed in (17). The values of the input attributes and the output attribute are normalized between 0 and 1. In the equation *a*_min⁡_ represents the minimum value for a in the column and *a*_max⁡_ represents the maximum value in the column and “*i*” symbolizes the column number [20–24] as follows:

(17) $$\begin{array}{c} \text{Normalized}\;\left(a_{i}\right)=\frac{\left(a_{i}-a_{\min}\right)}{\left(a_{\max}-a_{\min}\right)}. \end{array}$$

### 2.3. Classification Terms

 The classification accuracy used for the testing is calculated from the proportion of the number of patterns that are classified correctly to the number of all test patterns and expressed by (18) [21–24]. “Class. accur.” and “cor. class. pat.” are used as the abbreviations for “classification accuracy” and “correctly classified patterns”, respectively, in (18) [20, 21] as follows:

(18) $$\begin{array}{c} \text{Class}.\;\;\text{Accur}.\%=\left(\frac{\text{no}\;\text{of}\;\text{cor}.\;\text{class}.\;\text{pat}.}{\text{no}\;\text{of}\;\text{all}\;\text{test}\;\text{pat}.}\right)*100. \end{array}$$

Sensitivity and specificity are measures of performance used in classification systems. Sensitivity is calculated by the proportion of true positives to the sum of true positives and false negatives. This measures the ratio of the true positives in the sick people which are correctly identified. Sensitivity is also expressed as the ratio of positive (sick) classified patterns to the whole patterns (patients) with disease. The true positive term expressed that the patient has the disease and the classification (test) is positive. False positive explains that the patient does not have the disease but the test (classification) is positive. The true negative states that the patient does not have the disease and test is negative. The false negative expresses that the patient has the disease but the test or the classification is negative. 

 The reduced input attributes (colums) are tested in the neural network part of the developed software. In the classification procedure, the very risky and risky groups are accepted as positive (unhealthy or risky group), and the healthy group is accepted as the negatives. Sensitivity is expressed in (19). Specificity expresses the ratio of the number of true negative assessments to the sum of the numbers of false negatives and true positives. The specificity is shown in (20). In (19) and (20), the abbreviation “T. P.” depicts “True Positives” and “F. N.” expresses “False Negatives.” The abbreviations “T. N.”, “F. P.”, and “T. N.” express the “True Positives,” “False Positives,” and “True Negatives”, respectively. The number of the total test instances in the urological test data is expressed by *N*_test_ and is shown in (21). The terms used for the calculation of sensitivity, specificity, positive predictive value, and negative predictive value are given in Table 2 [25, 26].

(19) Sensitivity%  =Number  of  T.  P.Sum  of  Numbers  of  T.  P.  and  F.  N.∗100,

(20) Specificity%  =Number  of  T.  N.Sum  of  Numbers  of  T.  N.  and  F.  P.  ∗100,

(21) $$\begin{array}{c} N_{\text{test}}=\text{T}.\;\text{P}.+\text{T}.\;\text{N}.+\text{F}.\;\text{P}.+\text{F}.\;\text{N}. \end{array}$$

Positive predictive value and negative predictive value are two performance values of the tests and are calculated by using (22) and (23), respectively. “PPV” and “NPV” are used as the abbreviations for “positive predictive value” and “negative predictive value” in

(22) $$\begin{array}{c} \text{PPV}\%=\frac{\text{number}\;\text{of}\;\text{T}.\;\text{P}.}{\text{number}\;\text{of}\;\text{sum}\;\text{of}\;\text{T}.\;\text{P}.\;\text{and}\;\text{F}.\;\text{P}.}, \end{array}$$

(23) $$\begin{array}{c} \text{NPV}\%=\frac{\text{number}\;\text{of}\;\text{T}.\;\text{N}.}{\text{number}\;\text{of}\;\text{sum}\;\text{of}\;\text{T}.\;\text{N}.\;\text{and}\;\text{F}.\;\text{N}.}. \end{array}$$

The average classification sensitivity and specificity values were calculated and compared in (Section 3). The average classification sensitivity and specificity values are obtained by using the test data used in the neural network classifier by using the reducts.

## 3. Results and Discussions 

In the constructed FRSGSR, the urological database with 12 input variables is reduced and tested according the urological test database in the ANN part of the software. High average classification accuracy, sensitivity, specificity, PPV, and NPV results were obtained during the classification tests. The modified version of artificial selection algorithm was tested with the classical artificial selection algorithm and the roulette wheel selection mechanism. The computation time has been decreased averagely about 50% when compared with the roulette wheel selection mechanism and averagely 40% when compared with classical artificial selection algorithm when crossover and mutation rates were accepted as 50% and the percentages of good (best), intermediate, and worst solutions were accepted as 33%, 34%, and 33%, respectively. The designed software also supports the classical artificial selection and the best and worst percentages are accepted as 40% and 60% in the test procedure. In the selection process during the test procedures, the solution candidate taken from the middle point to the bottom also increases the performance by preventing the genetic algorithm system to be locked into some local solutions and helps the system for finding the reducts more rapidly. The system explores the reducts in 2 to 20 minutes of time depending upon the number of individuals in the population starting generations when modified artificial selection algorithm is used. The test operations are made by using Core2Quad 3.0 processor with 8 GB RAM. In the genetic algorithm based systems, locking to the local solutions is also a serious problem. These problems are solved by using the modified selection algorithm that forms the initial two generations randomly. The developed system prevents the memory errors by using the memory more efficiently. Most of reduction algorithms do not support the systems with high input spaces. In the FRSGR, attribute dependency value of rough sets methodology is used as the fitness value and the threshold value can be changed by the user of the interface. In the test procedure, a software based on attribute dependency reduction system that explores full combinations that does not contain genetic search strategy (all substes representing the attributes) is also constructed by using Delphi 7 programming language for comparing the performance with FRSGR. Attribute dependency reduction without genetic search strategy that explores full combinations supports the systems with 11 input variables and does not support the systems with 12 input variables or higher because when the systems with 12 variables are tested the allocated memory demand exceeds 899 MB and this situation gives rise to memory errors. The input number restriction and memory tests are made with the data set of 120 transactions (rows). In the developed FRSGR, the input number restriction problems are eliminated and the software supports the systems up to 100 input numbers. And the system is also tested with the constructed decision relative discernibility based reduction test system software using Delphi programming language. Decision based discernibiliy based attribute reduction system also has input number restrictions and supports maximum the data set with 12 input variable because this approach also demands extreme storage area.

 The decision relative discernibility matrix and function based reducing procedures and most of rough sets based reduction algorithms require high memory usages that give rise to memory errors and also the long computation times. The discernibility matrix and function based reducing software are also constructed by using Delphi programming language for the test procedure for comparing performance with the developed hybrid system. The discernibility matrix and function based attribute reducing software supports maximum 12 inputs when 120 transactions (rows) are used. When the test data with 15 input variables (uroflowmetric data) are tested with the decision relative discernibility matrix and function based system, the processing time exceeds 4 hours and exceeds the memory allocated by the operating system and causes memory errors. When testing the decision relative discernibility approach, in the task manager of the operating system, the Memory-Peak Working Set exceeds 860 MB (allocated memory in the task manager of operating system) that give rise to memory error. The average classification accuracy of decision relative matrix based approach is about 80% when the urological test data are used. In Table 3, average time demand and memory usage levels are shown according to the selection mechanism and the number of inputs and used approach. The abbreviation “Mod. Art. Sel.” denotes “modified artificial selection” and “art. sel.” shows “the artificial selection” in Table 3. The allocated memory peak working set denotes the assigned memory only to the software and by the operating system. Successful and satisfactory results were obtained during the reduction process. The FRSGR has the capability of scanning different numbered input spaces and searching property of exploring for the reducts in the desired ranges.

 The modified artificial selection algorithm version used in the software decreases computation times and prevents the genetic algorithm part to be locked to the local solution candidates. The software can be run for different threshold values (attribute dependency values calculated) and different number of attribute ranges. The number of the input variables of the medical system (twelve) has been reduced to the reducts with seven, six, and five elements. Some of the reducts found by the FRSGR are listed with the attribute dependency values in Table 4. These reducts are calculated when threshold attribute (feature) dependency value is accepted as 0.96 or higher. The threshold value used gives the opportunity for finding more number of reducts with high classification accuracy, sensitivity, and specificity when urological test data and ANN classifier are used.

 FRSGR finds the significant attributes of the medical risk degree determination system for the urological illnesses like urethral obstructions, urethral strictures, and the urological illnesses and determines the risk factor according to the urological measurements (uroflowmetric measurements and residual urine volume). The reducts that are named as *a*_1_, *a*_2_, and *a*_3_ denote maximum flow rate, average flow rate, and residual urine volume, respectively. The reduct that includes the inputs named as *a*_1_, *a*_2_, *a*_3_, *a*_5_, *a*_6_, *a*_11_, *a*_12_ expresses that significant (dominant) attributes named as maximum flow rate, average flow rate, residual urine volume, and the sampled flow rate values named as *a*_5_, *a*_6_, *a*_11_, *a*_12_ can be used for faster processing of urologic database.

 The processing times of the artificial neural network system for training procedure have been reduced averagely above 70% during the test operations made with the full and reduced medical data set. The full data set is the urological data set with 12 input variables and used in the test procedures.

 Decision relative discernibility function is expressed below (The decision based discernibility equation is abbreviated). “+” shows the union “∪” operator and “∗” shows the intersection operator in the discernibility function as follows:

(24) fd  =  (a1+a2+a3+a5+a6+a7+a8+a9+a10+a11) ∗(a1+a2+a3+a6+a8+a9+a10+a11+a12) ∗(a1+a2+a4+a5+a7+a8+a9+a10+a11) ∗(a1+a2+a3+a6+a7+a8+a9+a10+a11+a12) ∗(a1+a2+a3+a5+a6+a7+a8+a9+a10+a11) ∗(a1+a2+a4+a5+a6+a7+a8+a10+a11+a12) ∗(a1+a2+a3+a5+a6+a7+a8+a9+a10+a11) ∗(a1+a3+a5+a6+a8+a10+a11) ∗(a1+a3+a4+a8+a10) ∗(a1+a6+a8+a10+a11) ∗(a1+a3+a5+a7+a8+a10) ∗(a1+a2+a3+a4+a5+a6+a7+a8+a9+a10   +a11+a12) ∗(a1+a2+a3+a4+a6+a7+a9+a10+a11) ∗(a1+a2+a3+a5+a7+a8+a9+a10+a11+a12) ∗⋯ ∗(a1+a2+a4+a5+a9+a11+a12) ∗(a1+a2+a3+a4+a5+a8+a9+a12)=(a2+a4+a6+a9+a10+a11) ∗(a1+a2+a5+a6+a7+a8+a10) ∗(a1+a2+a5+a6+a7+a10+a11) ∗(a1+a4+a7+a8+a10) ∗(a3+a8+a10) ∗(a1+a4+a6+a9+a11+a12) ∗(a1+a5+a6+a8+a11) ∗⋯ ∗(a3+a5+a11) ∗(a1+a6+a7+a9) ∗(a2+a5+a7+a9+a11) ∗(a1+a2+a4+a6+a7+a10+a11) ∗(a1+a3+a4+a6+a11) ∗(a3+a11+a12) ∗(a8+a10+a11) ∗(a1+a9+a10) ∗(a5+a9+a10).

After the second simplification procedure the reducts obtained from the decision relative discernibility based approach are given below.

(25) fd=a1a2a3a8a10+a1a2a3a7a9a10+a1a2a3a5a8a12+a1a2a3a5a6a11a12+a2a3a4a6a10a12+a2a3a4a8a9a12+a2a3a4a9a11a12+a2a3a6a7a8a10+a2a3a6a7a10a11+a2a3a5a7a9a10+a2a3a7a8a9a10+a2a3a7a8a10a11+  a2a3a6a8a10a12+⋯+a3a7a9a10a11+a3a7a9a11a12+a3a8a9a10a12+a3a8a9a11a12+a1a3a5a8a11a12+a1a3a5a8a10a11+a1a3a5a8a10a12+a1a3a8a10a11a12+a1a3a7a8a10a12.

We have tested average classification accuracies of decision relative discernibility and Johnson reducer algorithms with the urological test data base that consists of 12 input variables that we have used in this study for testing procedure. The average classification accuracies of 80% and 55% have been obtained for the decision relative discernibility and Johnson reducer algorithm, respectively. The same database is used for FRSGR and the average classification accuracy obtained is above 95%. In addition, higher average sensitivity, specificity, positive and negative predictive values are obtained by using FRSGR. The average classification accuracies, sensitivities, specificities, PPV and NPV percentages of the reducts of FRSGR, decision relative discernibility, and Johnson Reducer are shown in Table 5. The average sensitivity and specificity percentage values of the reducts of FRSGR have been obtained as 97% and 93%, respectively. The positive predictive and negative predictive value of FRSGR is about 95%. As shown in Table 5, higher sensitivity, specificity, positive and negative predictive values have been obtained by using FRSGR and these values are higher than decision relative discernibility and Johnson reducer when tests were made with variable input ANN classifier using urological test data. 

 During the test operations of Johnson reducer of Rosetta software, the found reducts with 2 elements or higher are evaluated (average of the reducts of full and object related discernibility). 

 During the classification test procedures, the inputs included by the reducts are tested in the neural network classification part of the software of the FRSGSR. Some of the reducts that are found by the Johnson reducer system are expressed below

(26) $$\begin{array}{c} a_{3}a_{7}a_{9}a_{10}a_{11}\\ a_{1}a_{2}a_{9}\\ a_{9}a_{11}a_{12}\\ a_{1}a_{4}a_{9}\\ a_{2}a_{10}\\ \vdots \\ a_{3}a_{4}\\ a_{3}a_{10}\\ a_{2}a_{7}\\ a_{2}a_{11}. \end{array}$$

FRSGR has found the significant reduced number of input attributes with high classification accuracy, sensitivity, specificity, PPV and NPV values when compared with Johnson algorithm (average of the reducts of full discernibility and object related discernibility) and decision relative discernibility based reduction system.

## 4. Conclusions

 The extreme memory demand and input space restriction problems of most of rough sets based and feature reduction systems are solved by using the designed software which has also the capability of finding the reducts (reduced input attributes) more faster and efficiently. Different reducts can be obtained by the developed system according to the user defined attribute dependency parameter and changing this threshold level gives the opportunity to determine the quality of classification. High classification accuracy, sensitivity, specificity, positive and negative predictive values are obtained for FRSGR when ANN based classifier is used for testing procedure.

 Most of the reducts with high attribute (feature) dependency values include the inputs named as *a*_1_, *a*_2_, and *a*_3_. The input variables *a*_4_, *a*_5_, *a*_6_, *a*_7_, *a*_8_, *a*_9_,…, *a*_12_ (9 sampled uroflowmetric values) symbolize the flow rate measurements in the period of *T*/4 and 3*T*/4. The obtained reducts show the significance of the maximum flow rate, average flow rate and residual urine volume measurements and some of the sampled flow rate values. The input medical data have been classified according to the obtained reducts with high performance and accuracy when compared with the tested algorithms. The proposed modified artificial selection algorithm prevents the genetic algorithm system to be locked into some points and helps the algorithm to find the results more effectively and rapidly by exploring the solution space with high performance. FRSGR supports high dimensional input spaces and more input variables when compared with the tested systems. The significant attributes of the medical system (urological database) have been determined by the FRSGR for faster training and processing with soft computing techniques.

## Figures and Tables

**Table 1:** Some of the transactions in the medical (urological) database.

	*a*_1_	*a*_2_	*a*_3_	*a*_4_	*a*_5_	*a*_6_	*a*_7_	*a*_8_	*a*_9_	*a*_10_	*a*_11_	*a*_12_	*d*
1	1	1	2	1	1	1	1	1	1	1	1	1	1
2	1	1	3	1	1	1	1	1	1	1	1	1	1
3	1	1	2	1	1	1	1	2	1	1	1	1	1
4	2	1	1	2	1	2	1	1	1	2	1	1	1
5	2	1	1	1	1	1	2	1	1	2	1	2	1
6	2	1	2	1	2	1	1	1	1	2	1	1	1
7	2	1	2	1	1	1	2	2	2	1	1	1	1
8	3	3	1	3	3	3	4	3	3	3	3	3	3
9	3	3	1	3	3	3	3	3	3	3	3	3	3
10	2	1	1	2	1	2	1	1	1	2	1	1	1
11	4	3	1	3	3	3	4	3	3	3	3	3	3
12	2	1	1	2	1	1	1	2	2	1	1	1	1
13	2	1	2	2	1	1	1	1	2	1	1	2	1
14	3	3	1	3	3	3	3	3	4	3	3	3	3
15	2	1	2	2	1	1	1	1	2	1	1	2	1

**Table 2:** The terms used for the calculation of sensitivity, specificity, NPV, and PPV.

Diagnostic Test or Classification	Disease (Positive) (Patients with Urological Disease or Risky Patients)	Disease Negative (Patients without Urological Disease or Healthy Patients
Test Positive	True Positive (T. P.)	False Positive (F. P.)
Test Negative	False Negative (F. N.)	True Negative (T. N.)

The Column Total	(T. P.) + (F. N.)	(F. P.) + (T. N.)

**Table 3:** Average time interval and memory usage levels of tested system softwares.

	Tested System		Number of Inputs	Time (average)	Allocated Memory Peak Working Set (MB), (average res.)
		Modified Artificial Selection	12	2–20 min.	70–250 MB
1	FRSGR	Artificial Selection	12	3.5–33 min.	65–300 MB
		Roulette Wheel Selection	12	4–35 min.	75–320 MB
		Modified Artificial Selection	15	4–30 min.	75–320 MB

			12	70 min.	380 MB
2	Decision Relative Discenibility				
			15	Exceeds 4 Hours	Exceeds 860 MB and causes memory error (insufficient memory)

3	Attribute dependency reduction without genetic search		12	Exceeds 2 Hours	Exceeds 899 MB and causes memory error

**Table 4:** Some of the reducts found by the developed FRSGR.

Element Number	The Reducts	Attribute Dependency Value
7	*a*_1_ *a*_2_ *a*_3_ *a*_5_ *a*_6_ *a*_11_ *a*_12_	1
	*a*_2_ *a*_3_ *a*_4_ *a*_8_ *a*_9_ *a*_11_ *a*_12_	1

	*a*_1_ *a*_2_ *a*_3_ *a*_7_ *a*_9_ *a*_10_	1
	*a*_1_ *a*_2_ *a*_3_ *a*_5_ *a*_8_ *a*_12_	1
	*a*_1_ *a*_3_ *a*_4_ *a*_5_ *a*_8_ *a*_10_	1
	*a*_2_ *a*_3_ *a*_5_ *a*_8_ *a*_9_ *a*_12_	1
	*a*_1_ *a*_3_ *a*_6_ *a*_8_ *a*_9_ *a*_10_	1
6	*a*_1_ *a*_2_ *a*_3_ *a*_7_ *a*_9_ *a*_10_	1
	*a*_1_ *a*_2_ *a*_3_ *a*_5_ *a*_8_ *a*_12_	1
	*a*_1_ *a*_3_ *a*_4_ *a*_5_ *a*_8_ *a*_10_	1
	*a*_2_ *a*_3_ *a*_5_ *a*_8_ *a*_9_ *a*_12_	1
	*a*_1_ *a*_3_ *a*_6_ *a*_8_ *a*_9_ *a*_10_	1
	*a*_2_ *a*_3_ *a*_7_ *a*_8_ *a*_9_ *a*_10_	1

	*a*_2_ *a*_3_ *a*_4_ *a*_6_ *a*_8_ *a*_12_	0.975
	*a*_2_ *a*_3_ *a*_4_ *a*_8_ *a*_10_ *a*_12_	0.967
6	*a*_2_ *a*_3_ *a*_4_ *a*_8_ *a*_10_ *a*_11_	0.975
	*a*_2_ *a*_3_ *a*_4_ *a*_7_ *a*_9_ *a*_11_	0.983
	*a*_2_ *a*_3_ *a*_4_ *a*_8_ *a*_10_ *a*_11_	0.975

5	*a*_1_ *a*_2_ *a*_3_ *a*_8_ *a*_10_	1

**Table 5:** Classification accuracies, sensitivities, specificities, PPV and NPV of FRSGR, decision relative discernibility, and Johnson reducer.

Tested System Software	Average Classification Accuracy (%)	Average Sensitivity (%)	Average Specificity (%)	PPV (%)	NPV (%)
(1) FRSGR	95	97	93	95	95
(2) Decision Relative Discernibility	80	82	78	84	74
(3) Johnson Reducer (Rosetta) (Full and Object Related Discernibility)	55	52	60	66	45
